# PC-CS-YOLO: High-Precision Obstacle Detection for Visually Impaired Safety

**DOI:** 10.3390/s25020534

**Published:** 2025-01-17

**Authors:** Jincheng Li, Menglin Zheng, Danyang Dong, Xing Xie

**Affiliations:** 1 School of Artificial Intelligence and Computer Science, Nantong University, Nantong 226019, China; 2330110412@stmail.ntu.edu.cn (J.L.); 2230110478@stmail.ntu.edu.cn (M.Z.); danyangdong@stmail.ntu.edu.cn (D.D.); 2 Engineering Training Center, Nantong University, Nantong 226019, China

**Keywords:** YOLO11, deep learning, object detection, visually impaired, PC-CS-YOLO

## Abstract

The issue of obstacle avoidance and safety for visually impaired individuals has been a major topic of research. However, complex street environments still pose significant challenges for blind obstacle detection systems. Existing solutions often fail to provide real-time, accurate obstacle avoidance decisions. In this study, we propose a blind obstacle detection system based on the PC-CS-YOLO model. The system improves the backbone network by adopting the partial convolutional feed-forward network (PCFN) to reduce computational redundancy. Additionally, to enhance the network’s robustness in multi-scale feature fusion, we introduce the Cross-Scale Attention Fusion (CSAF) mechanism, which integrates features from different sensory domains to achieve superior performance. Compared to state-of-the-art networks, our system shows improvements of 2.0%, 3.9%, and 1.5% in precision, recall, and mAP50, respectively. When evaluated on a GPU, the inference speed is 20.6 ms, which is 15.3 ms faster than YOLO11, meeting the real-time requirements for blind obstacle avoidance systems.

## 1. Introduction

Efficient blind obstacle detection systems are vital for visually impaired individuals. According to a report [1] released by the World Health Organization on 14 October 2021, at least 2.2 billion people globally have some form of vision impairment, with approximately 1 billion suffering from moderate to severe visual impairment or blindness. People with visual impairments face many challenges when traveling, such as difficulty identifying roads, trouble boarding transportation, and lack of companions for travel. Furthermore, issues such as the obstruction of tactile paving and inadequate accessibility facilities further exacerbate the difficulties faced by individuals with visual impairments. Despite the efforts made by governments and international organizations to implement policies [2,3] aimed at improving travel conditions for people with visual impairments, they still encounter numerous challenges in practice. These difficulties not only affect their quality of life but also limit their ability to participate in social activities. Therefore, it is crucial to adopt innovative technologies to address the travel challenges of people with visual impairments, especially in terms of recognizing and avoiding obstacles.

People with visual impairments often believe that obstacles in the external environment have the greatest impact, especially those around sidewalks. Therefore, effectively identifying and avoiding obstacles, particularly static and dynamic ones, becomes a key challenge for the safe travel of individuals with visual impairments. Static obstacles, such as traffic cones and damaged roads, are often hard to detect and may cause individuals with visual impairments to trip or become injured. Dynamic obstacles, such as pedestrians and vehicles, are more complex as their position, and behavior may change according to the environment, which increases the difficulty of avoidance. To address these issues, the development of new obstacle avoidance technologies and algorithms is crucial in providing safe and reliable travel solutions for individuals with visual impairments.

In recent years, technologies based on computer vision [4], deep learning [5], and machine learning have been widely applied in navigation and obstacle avoidance systems for individuals with visual impairments. Ross [6] proposed the use of wearable computing devices as virtual environmental interfaces to help visually impaired individuals perceive their surroundings. Bourbakis et al. [7] developed the Tyflos smart assistant system, which uses vision cameras to capture environmental information and interacts with users through voice feedback. Lee et al. [8] proposed a navigation system based on RGB-D cameras, which provides indoor and outdoor navigation assistance through tactile feedback. However, current obstacle detection technologies still face challenges, primarily including low detection accuracy, poor real-time performance, and difficulty in reliably identifying obstacles in complex environments. Therefore, further improving the accuracy and real-time capabilities of obstacle detection has become critical to addressing the obstacle avoidance needs of visually impaired individuals.

To address these issues, we propose an improved vision model that combines YOLO11, Partial Convolution-based Feed-forward Network (PCFN [9]), and Cross-Scale Attention Fusion (CSAF). The contributions of this paper are summarized below:

1. This paper evaluates the potential of YOLO11 as an assistive method for obstacle detection in blind navigation. YOLO11 demonstrates improved performance in handling multiple objects in complex scenes, particularly for obstacles that are overlapping or densely distributed, offering superior detection capabilities.

2. We propose a novel PC-CS-YOLO neural network, which outperforms YOLO11 in terms of precision, recall, and mAP by 2%, 3.9%, and 1.5%, respectively, on a specific dataset. The network improves the backbone architecture by employing a PCFN (Partial Convolution-based Feed-forward Network) mechanism to reduce computational redundancy. Additionally, the integration of the CSAF (Cross-Scale Attention Fusion) mechanism enhances the network’s robustness in multi-scale feature fusion, thereby achieving superior performance.

3. Evaluation on an NVIDIA RTX 4060 demonstrates an inference speed of only 20.6 ms, which is 15.3 ms faster than YOLO11, meeting the real-time requirements of blind navigation systems.

Through these improvements, our system is able to provide more accurate and reliable obstacle detection services in various complex environments, particularly handling both dynamic and static obstacles more effectively. This system offers strong support for the safe navigation of visually impaired individuals, helping them walk more confidently in their environment while avoiding potential risks and dangers.

## 2. Related Work

The YOLO series models, known for their simple structure and high accuracy, have been widely applied in obstacle detection. Erdaw et al. [10] proposed a real-time obstacle detection method based on YOLOv2, which effectively helps visually impaired individuals identify obstacles such as potholes and trash bins. Alsultan et al. [11] used the YOLOv7 model for the real-time recognition of environmental objects. Chaudhary et al. [12] developed a device based on YOLOv3, which not only detects obstacles but also provides distance estimates, helping visually impaired individuals navigate more safely. However, these models still face challenges in detecting multiple obstacles, particularly small ones, with insufficient accuracy. Multi-object detection and small object detection remain significant challenges for YOLO models.

Zhang et al. proposed an innovative lightweight convolutional neural network architecture that combines inverse residual structures and the Coordinate Attention (CA) mechanism, designing the Coordinate Attention Mobile (CAM) network. This method enhances small object detection accuracy through multi-scale feature extraction while reducing computational complexity, making it suitable for resource-limited embedded devices [13]. Kisantal proposed an improved method that enhances small object detection accuracy by oversampling images containing small objects and augmenting them through multiple copy-pasting of small objects [14]. Additionally, Zhang et al. optimized the Darknet network by adopting depthwise separable convolutions and the channel attention (ECA) mechanism, significantly improving detection performance while reducing the computational parameters [15]. This method effectively reduces computation and storage overhead while maintaining high accuracy. Shi et al. proposed FocusDet, an innovative method for small object detection, which combines the STCF-EANet backbone network and the Bottom Focus-PAN feature fusion module [16]. In the field of remote sensing imagery, Zhang et al. proposed the EB-Net model, which uses DSFormer to achieve efficient feature extraction, balancing both accuracy and computational efficiency [17].

Kumar and Jain [18] used the YOLOv5 model for obstacle detection; however, it shows limitations in detecting small objects and meeting real-time requirements. Particularly in complex environments, the model struggles with identifying smaller obstacles accurately, leading to a higher risk of missed detections. Additionally, although the detection speed has been improved, it still falls short of real-time requirements, making it difficult for visually impaired individuals to respond promptly to changes in their surroundings. Similarly, Atitallah et al. [19] proposed an improved YOLOv5 model that incorporates the CSPNet (Cross Stage Partial Network) as the backbone network for assisting visually impaired individuals in obstacle detection. However, the model still exhibits shortcomings in small object detection, complex scene adaptation, and real-time performance, such as missed detections due to the occlusion of small objects and limited recognition capability for dynamic obstacles. Boussihmed et al. [20] introduced a lightweight YOLO model based on TinyML for pedestrian obstacle detection. Nonetheless, it still faces challenges in small object detection. Due to the reduction in model parameters, the algorithm tends to miss small obstacles such as traffic cones and pavement defects in complex scenes. To address these issues, we propose the PC-CS-YOLO model, which reduces redundant calculations while improving the detection accuracy of small obstacles and the adaptability to complex environments.

## 3. Materials and Methods

### 3.1. Data

#### 3.1.1. Data Types

During the mobility of visually impaired individuals, the timely identification and avoidance of obstacles is crucial [21]. To assist blind individuals in effectively avoiding obstacles along their travel route, we study several typical obstacles, including cars, pedestrians, traffic cones, and surface defects. Among these, traffic cones and surface defects are non-living entities that cannot actively avoid the blind, making it even more essential to design a detection system that can accurately identify these obstacles. Table 1 outlines the recognition rules for typical types of obstacles.

#### 3.1.2. Data Preprocessing

Data preprocessing in this study includes four main steps: image collection, filtering, labeling, and data enhancement.

Image Collection: This study utilizes a publicly available road obstacle dataset, which contains 8120 images in JPG format. These images cover a variety of road environments and obstacle types, providing a rich set of training data for the object detection model.

Filtering: To ensure the high quality of the dataset, we performed a rigorous filtering process on the original images. First, we excluded any images with significant occlusions or poor quality. After this filtering step, 6464 high-quality images remained, which are suitable for training the model.

Labeling: The original dataset was annotated in JSON format. However, to make it compatible with the YOLO model, we converted the annotations to the COCO dataset format [22]. During this process, we preserved all annotation information for each image and ensured the accuracy and consistency of the bounding boxes. The 6464 images and their corresponding annotation files were then split into training, validation, and test sets using a 7:2:1 ratio. It is important to note that each image may contain multiple obstacle instances, and the number of instances of each obstacle varies by class as shown in Table 2.

Data enhancement: For data augmentation, this study adopted the Mosaic Data Augmentation (MDA) [23] method used in the YOLO model. This method incorporates five augmentation techniques: image stitching, flipping, cropping, random rotation, and HSV color space enhancements [24]. These augmentation methods allow us to generate a variety of image variants, helping the model better adapt to various scenarios and changes.

### 3.2. Our Model

#### 3.2.1. PCFN

When blind individuals walk on the street, they navigate through a complex background and are likely to encounter multiple obstacles of varying sizes simultaneously. This presents significant challenges for real-time obstacle detection, as it often leads to a substantial decrease in detection accuracy and a sharp increase in computation time. Convolutional Neural Networks (CNNs) [25] utilize a set of learnable filters (convolutional kernels) that slide across the entire input image, performing the same mathematical operations on each local region. The operations are identical for each pixel location, which inevitably results in a considerable loss of information. This approach also reduces the model’s ability to adapt to objects of different sizes and types, limiting its generalization capability. To address these shortcomings, we employ a partial convolutional feed-forward network, based on partial convolutions, to further enhance feature representation and improve performance.

**Figure 1:**
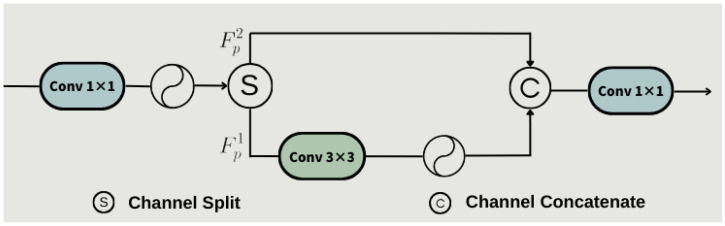
The structural diagram of the PCFN module.

Figure 1 illustrates the operation of the PCFN, where a 1 × 1 convolution with Gaussian Error Linear Unit (GELU) [26] activation is first applied to the expanded hidden space to enable cross-channel interactions. The hidden features are then divided into two parts, $\left\{F_{\rho }^{1},F_{\rho }^{2}\right\}$, and a 3 × 3 convolution followed by a GELU activation is applied to $F_{\rho }^{1}$ to capture the local contextual information. After processing, the features $F_{\rho }^{1}$ and $F_{\rho }^{2}$ are concatenated and passed through another 1 × 1 convolution to further mix the features and reduce the hidden channels back to the original input dimension. This process is formally defined as follows:

(1) $$\left\{F_{\rho }^{1},F_{\rho }^{2}\right\}=S\left(\phi \left({\mathrm{Conv}}_{1\times 1}\left({∥F_{\rho }∥}_{2}\right)\right)\right),$$

(2) $${\hat{F}}_{\rho }={\mathrm{Conv}}_{1\times 1}\left(C\left(\phi \left({\mathrm{Conv}}_{3\times 3}\left(F_{\rho }^{1}\right)\right),F_{\rho }^{2}\right)\right).$$

where $F_{\rho }^{1}$ is a tensor of dimension $H\times W\times \frac{C}{2}$, and $F_{\rho }^{2}$ is a tensor of dimension $H\times W\times \frac{3C}{2}$. The symbol C(·) denotes the concatenation operation, while ${Conv}_{3\times 3}\left(\cdot \right)$ represents a 3 × 3 convolutional layer.

The PCFN enhances the features extracted by the network through cross-channel and spatial interactions [27]. By reducing computational redundancy while maintaining efficient feature fusion, the PCFN model improves the accuracy of obstacle detection.

#### 3.2.2. CSAF

Obstacle detection for blind pedestrians often faces challenges in complex environments, especially when the features of small targets are weakened. Small obstacles, such as traffic cones and pavement defects, are often overlooked or inaccurately detected due to their small size.

Existing YOLO-based models typically use traditional Feature Pyramid Networks (FPNs) [28], which only fuse features from adjacent layers, leading to information loss. This issue is particularly pronounced when processing obstacles of varying scales in complex backgrounds. Therefor, we propose a Cross-Scale Attention Fusion (CSAF) mechanism, which effectively fuses features across different scales to capture multi-scale global contextual information. This approach helps reduce the interference from background noise and improves detection accuracy.

**Figure 2:**
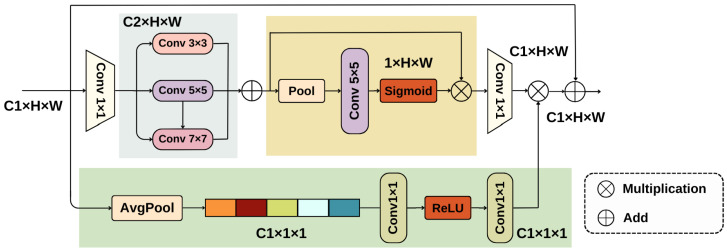
The structural diagram of the CSAF mechanism.

The CSAF mechanism is accomplished through two modules: the spatial refinement path module and the channel aggregation path module. Figure 2 provides a detailed representation of this layout.

In the spatial refinement path of the CSAF mechanism, the input feature map’s channel dimensions are first reduced from $C_{1}$ to $C_{2}$ using a 1 × 1 convolution, where $C_{2}=C_{1}\times \alpha$ (with α being a factor less than 1, used for dimensionality reduction). This operation helps to compress features along the channel dimension, thereby reducing computational complexity. Next, the CSAF mechanism performs multi-scale feature fusion using convolutional kernels of different sizes (e.g., 3 × 3, 5 × 5, and 7 × 7) to capture spatial information at various scales [29]. This process effectively preserves the features of small targets and enhances the model’s ability to detect obstacles of different sizes. Subsequently, the fused features are aggregated using both average pooling and max pooling [30], where the former captures global information and the latter extracts the most prominent local features. Finally, the aggregated features are passed through a sigmoid activation function [31] to generate a spatial attention map, which is then element-wise multiplied with the original features. This further refines the spatial attention, enabling the model to focus more on important spatial regions. Here is the specific formula:

(3) $$\sigma \left(\chi \right)=\frac{1}{1+e^{-\chi^{2}}}$$

(4) Channel Attention Map=ReLU(1×1convolutions on GlobalAvgPool(F))

(5) ReLU(x)=max(0,x)

where Equation (3) is the mathematical expression of the sigmoid function, which maps inputs to the range (0,1) and has an S-shaped graph. Equations (4) and (5) use global average pooling to reduce dimensions, then generate channel attention maps through a 1 × 1 convolution followed by a ReLU activation function.

The CSAF mechanism achieves multi-scale spatial information fusion [32] by summing convolutions with different kernel sizes and performing a series of spatial feature aggregation operations [33]. Additionally, global average pooling, convolution, and activation functions are used to generate a channel attention map, which is then combined with the spatially refined feature map to integrate multi-scale information along the channel dimension [34]. This approach effectively identifies obstacles of various sizes in complex backgrounds, providing accurate obstacle avoidance decision support for blind pedestrians.

#### 3.2.3. PC-CS-YOLO

Due to its optimal balance between speed and accuracy, the YOLO series models are widely used in various recognition tasks. In this study, we adopt the latest version, YOLO11, as the backbone of our model. YOLO11 consists of a backbone network, a neck network, and a detection head. The backbone integrates the C3K2 module [35], which is designed to select the most suitable method for feature extraction based on different requirements and scenarios. The neck network introduces the C2PSA module after the SPPF module, which consists of two convolutional layers and a multi-head self-attention module. This module aims to enhance feature extraction by leveraging the multi-head attention mechanism and feed-forward neural networks. After the convolution, the output is forwarded to the detection head, where the final classification and regression tasks are solved.

In the obstacle detection task, the feed-forward neural network (FFN) [36] in the C2PSA module is used to extract image features. Since each layer in the FFN is fully connected, the signal propagates from the input layer to the output layer without feedback connections, which leads to lower computational efficiency when processing high-dimensional data. To address this issue, we replace the FFN with a Partially Convolutional Feed-forward Network (PCFN) and integrate it with the C2PSA module to create the C2PSA-PCFN module. Furthermore, we combine the PCFN with the C3K2 module, replacing its convolutional layers, to form the C3K2-PCFN module. This modification reduces computational redundancy while maintaining efficient feature fusion.

**Figure 3:**
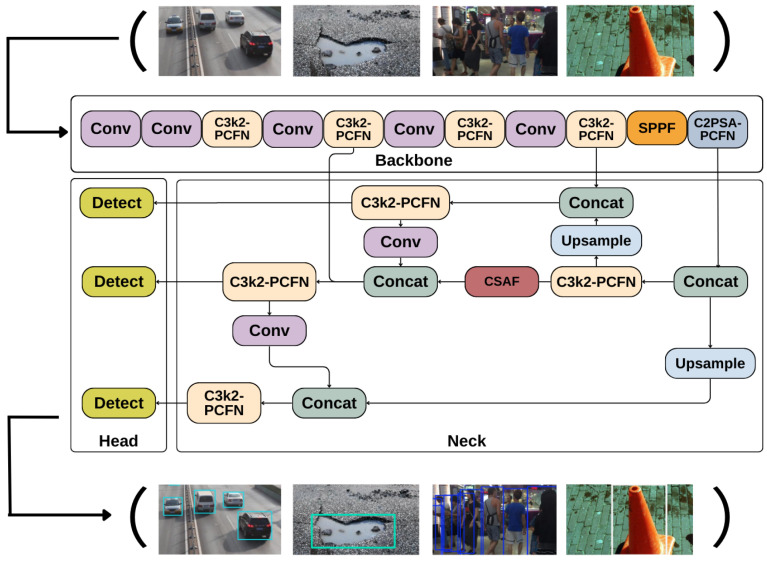
The structural diagram of PC-CS-YOLO.

On the other hand, to address the potential limitations in feature extraction or insufficient multi-scale information fusion during cross-layer feature concatenation in YOLO11, we introduce the CSAF mechanism after the contact layer in the neck section. This enhancement helps improve the model’s detection accuracy for obstacles of varying sizes in complex backgrounds. The structure of PC-CS-YOLO is shown in Figure 3.

## 4. Results and Analysis

### 4.1. Experiment Environment

This PC-CS-YOLO network is trained on a single NVIDIA GeForce RTX 4060 graphics card (NVIDIA, Santa Clara, CA, USA) with 8 GB of memory. The experimental environment utilizes Anaconda as the Python package management tool and is equipped with the PyTorch deep learning framework. All experiments are conducted on a 64-bit Windows 11 system with CUDA 12.1, Python 3.10, and PyTorch 2.0.1. Proper hyperparameter configuration is crucial in the model training process. Table 3 lists the specific hyperparameters used in this study.

### 4.2. Evaluation Metrics

In order to evaluate the proposed method, we use accuracy, recall [37], and mean Average Precision (mAP) [38] as evaluation metrics to measure model performance. The definitions of each metric are as follows:

(6) $$\mathrm{Precision}=\frac{\mathrm{TP}}{\mathrm{TP}+\mathrm{FP}}$$

(7) $$\mathrm{Recall}=\frac{\mathrm{TP}}{\mathrm{TP}+\mathrm{FN}}$$

(8) $$\mathrm{AP}=\int_{0}^{1}P\left(R\right)\;dR$$

(9) $$\mathrm{mAP}=\frac{1}{C}\sum_{i=1}^{C}{\mathrm{AP}}_{i}$$

True Positives (TP) represent the number of instances correctly predicted as positive by the model; False Positives (FP) indicate the number of negative instances incorrectly predicted as positive; False Negatives (FN) refer to the number of positive instances mistakenly classified as negative by the model. The precision is calculated as TP divided by the sum of TP and FP, measuring the accuracy of the model’s positive predictions. Recall is computed as the ratio of TP to the sum of TP and FN, reflecting the model’s ability to identify positive samples [39].

The Average Precision (AP) represents the area under the Precision–Recall (P-R) curve [40], which can be obtained by integrating this curve. The constant CCC denotes the number of classes. In this study, there are four classes: vehicles, pedestrians, traffic cones, and road surface defects. For multi-class problems, AP is calculated separately for each class, and the mean of these values is taken to obtain mAP, thus accommodating the unique P-R curve of each class.

### 4.3. Ablation Experiment

In this section, some ablation experiments are conducted to evaluate the impact of the PCFN structure and CSAF module on the obstacle detection performance of the YOLO11 model. Higher precision indicates reduced model inaccuracies, thereby minimizing the inconveniences caused by prediction errors for blind travelers [41]. An improvement in recall reflects the comprehensiveness of obstacle detection, reducing the risk associated with missed detections.

As shown in Table 4, data analysis reveals that integrating only the PCFN structure into the backbone results in a 1.7% increase in precision, a 1.4% increase in recall, and a 0.3% increase in mAP. This demonstrates that PCFN successfully enhances the model’s recognition precision and recall by improving the feature extraction capabilities and reducing computational redundancy, even though the improvement in mAP is modest. Furthermore, introducing the CSAF mechanism into the neck results in a 1.4% increase in precision, a 3.8% increase in recall, and a 0.5% increase in mAP. Although the precision gain from the CSAF mechanism is relatively small, it significantly improves recall by enhancing the model’s multi-scale feature fusion capability, enabling the detection of obstacles of various sizes.

Upon synergizing these modules, the PC-CS-YOLO model demonstrates notable improvements over YOLO11 across all evaluation metrics, with a 2.0% increase in precision, a 3.9% increase in recall, and a 1.5% increase in mAP. The design of PCFN and CSAF achieves a harmonious improvement in both precision and recall. The results validate the effectiveness of these two modules in optimizing the backbone and neck structures of YOLO11, thereby enhancing the model’s obstacle detection capabilities.

In blind obstacle detection systems, the processing time of the network must meet real-time requirements. In order to obtain fair statistics, all measurements are performed on the same single NVIDIA RTX 4060 card using the obstacle dataset. We perform experiments on a detection task involving 756 images and measure the inference time, ultimately calculating the average inference time per image. The results are presented in Table 5. As depicted in Table 5, the proposed PC-CS-YOLO model achieves an average per-image inference time improvement of 15.3 ms compared to the YOLO11 model. Specifically, the partial convolution mode utilized by the PCFN module significantly reduces computational redundancy, leading to enhanced efficiency.

### 4.4. Comparative Analysis of Model Performance

**Figure 4:**
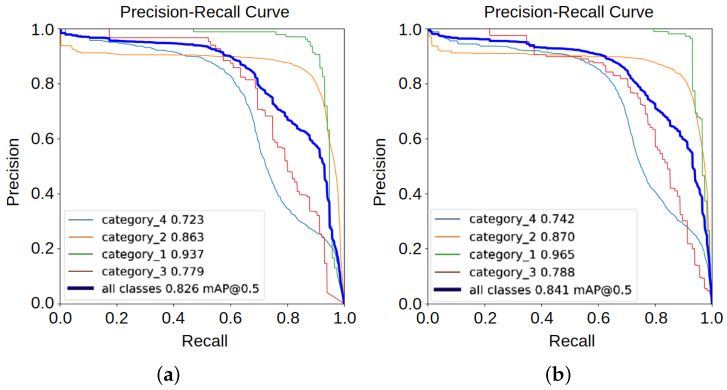
P-R curve: (**a**) YOLO11; (**b**) PC-CS-YOLO.

We plot the Precision–Recall (P-R) curves of YOLO11 and PC-CS-YOLO, with precision on the x-axis and recall on the y-axis as shown in Figure 4. The area under each P-R curve represents the Average Precision (AP), with a larger area indicating a higher AP value. This figure shows the improvements in AP for different categories, where category_1, category_2, category_3, and category_4 represent vehicles, pedestrians, traffic cones, and road surface defects, respectively.

Categories with slight AP changes worth noting include pedestrians. In contrast, the AP differences for cars and traffic cones are more significant, increasing by 2.8% and 0.9%, respectively. Particularly noteworthy is the improvement in road surface defects, with an initial mAP of 72% rising to 74.2% after using PC-CS-YOLO. Road surface defects are small and prone to being missed, and the increase in mAP can be attributed to the PCFN and its enhancement of the model’s feature extraction capability, along with the CSAF and its improvement in multi-scale feature fusion.

**Figure 5:**
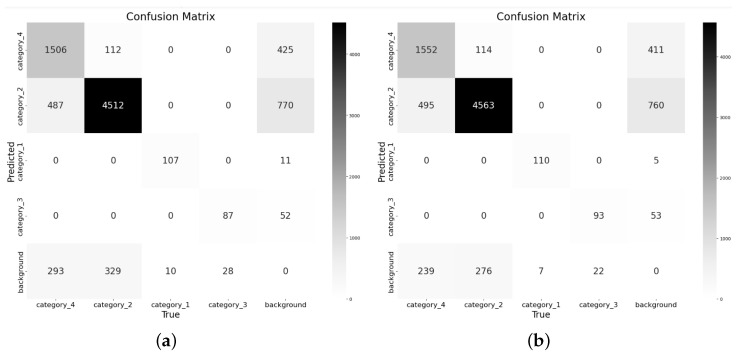
Confusion matrix: (**a**) YOLO11; (**b**) PC-CS-YOLO.

The 2D confusion matrix [42] provides an intuitive comparison of the predicted results with actual results to evaluate the effectiveness of the behavior recognition model. Figure 5 shows the confusion matrices of YOLO11 and PC-CS-YOLO on the test dataset. In comparison, PC-CS-YOLO demonstrates higher accuracy in detecting all four types of obstacles. Specifically, PC-CS-YOLO correctly identifies more instances of cars, pedestrians, traffic cones, and road surface defects, with increases of 3, 51, 6, and 46 instances, respectively.

### 4.5. Comparative Analysis with Other Models

To validate the effectiveness and superiority of PC-CS-YOLO in obstacle recognition, we compare it with several prominent models in the field of object detection. The evaluation includes well-known models such as EfficientDet, Faster R-CNN, YOLOv5, YOLO11, and D-FINE [43]. All models are tested using the same dataset and evaluation criteria, with the results presented in detail in Table 6.

The analysis in Table 6 shows that PC-CS-YOLO outperforms EfficientDet, YOLO11 and D-FINE in both precision and recall. Although slightly lower in precision compared to EfficientDet, PC-CS-YOLO excels in recall and mAP, achieving 79.7% and 84.1%, respectively, which surpasses most other models. In terms of precision, recall, and mAP, PC-CS-YOLO significantly outperforms YOLOv5, fully demonstrating the superiority of the model.

### 4.6. Model Visualization

**Figure 6:**
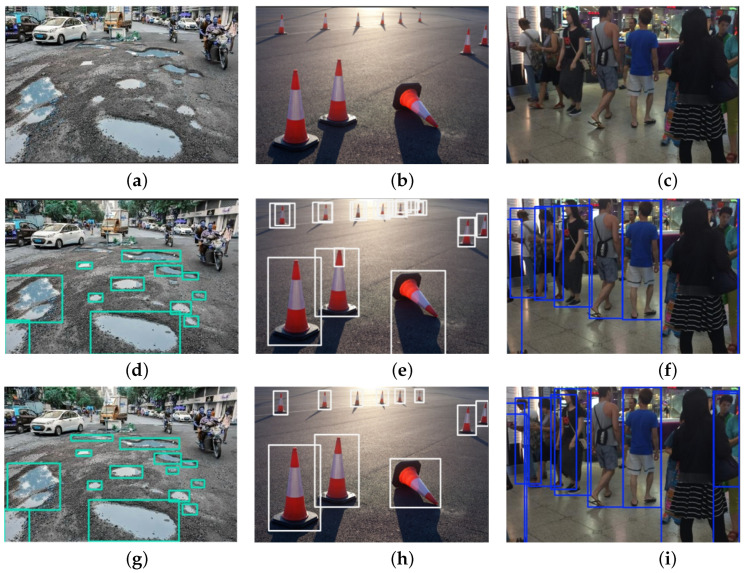
Visualization map: (**a**–**c**) raw image; (**d**–**f**) YOLO11; (**g**–**i**) PC-CS-YOLO.

To further validate our model’s performance in recognizing obstacles of various sizes and in complex scenes, we select multiple images reflecting different obstacle types and scene complexities. We conduct a comparative analysis between the improved PC-CS-YOLO model and the original YOLO11 baseline. Through visualization, we are able to intuitively evaluate the model’s recognition effectiveness and interpretability. Figure 6 illustrates the recognition results, with Figure 6a–c showing the original image, Figure 6d–f presenting the recognition results of YOLO11, and Figure 6g–i displaying the results of PC-CS-YOLO.

In the first column of Figure 6, YOLO11 fails to detect small road surface defects in the top-left corner of the Figure 6d, while PC-CS-YOLO accurately detects these defects. In the second column, YOLO11 is affected by the lighting conditions and mistakenly detects some traffic cones multiple times, particularly in the bottom-right corner of the Figure 6e, where it wrongly classifies a shadow as an independent traffic cone. PC-CS-YOLO does not make such errors and successfully identifies each traffic cone. In the last column of Figure 6, YOLO11 fails to detect occluded pedestrians, whereas PC-CS-YOLO accurately marks each pedestrian even under occlusion. In summary, compared to YOLO11, PC-CS-YOLO demonstrates better adaptability in complex environments, significantly improving detection accuracy and proving the robustness of the proposed model.

## 5. Discussion

The outstanding performance of the PC-CS-YOLO model in obstacle detection demonstrates its potential to provide precise obstacle avoidance support for visually impaired individuals [44], particularly excelling in handling small and densely distributed obstacles. By incorporating the Partial Convolutional Feed-forward Network (PCFN) and the Multi-Scale Attention Fusion (CSAF) modules, PC-CS-YOLO significantly improves detection accuracy and recall, with these results validated on a dataset of four typical obstacles.

Traditional obstacle detection techniques, such as the YOLO series models, often face challenges in terms of accuracy and real-time performance when detecting small objects or multiple obstacles in complex scenes. In this study, the YOLO11 architecture is enhanced with the addition of the PCFN and CSAF modules, which improve feature extraction and multi-scale information fusion. The primary role of the PCFN module is to address the significant decline in accuracy and the increased computation time when detecting multiple obstacles of varying sizes in complex backgrounds, while the CSAF module further optimizes the model’s ability to focus on multi-scale features, especially for small objects.

Experimental results show that PC-CS-YOLO outperforms YOLO11 by 2%, 3.9%, and 1.5% in terms of accuracy, recall, and mAP, respectively. When evaluated on a GPU, the inference speed is only 20.6 ms, improving computation time by 15.3 ms compared to YOLO11, thus meeting the real-time requirements of a blind navigation system.

Although our work has made some progress, there are still some limitations. Firstly, the model has not been specifically trained to recognize special obstacles, so its performance is suboptimal when identifying obstacles with unique shapes or materials. To improve the model’s recognition capability, future work could address this limitation by incorporating more diverse training data. Secondly, our model currently struggles with detecting objects when parts of their features are occluded in highly complex environments. To address this limitation, we plan to enhance our model’s capability in occlusion detection, which will help us improve object detection performance under these challenging conditions.

Furthermore, this study primarily focuses on detecting common obstacles, but future work aims to extend the model to detect more specialized obstacles. This will lay a solid foundation for developing safer and more reliable obstacle avoidance systems, effectively addressing the navigation needs of visually impaired individuals in their daily lives. To achieve this, we need to increase the diversity of the training data to enhance the model’s recognition capabilities.

## 6. Conclusions

This study aims to identify obstacles encountered by visually impaired individuals during their travel and provide decision support for obstacle avoidance. To achieve this, we propose the PC-CS-YOLO detection model based on the YOLO11 framework. First, to address the issues of significantly reduced accuracy and increased computation time when detecting multiple obstacles of varying sizes in complex backgrounds using the YOLO model, we integrate the PCFN module into the backbone network and combine it with the C2PSA and C3K2 modules to construct the C2PSA-PCFN and C3K2-PCFN modules. Through cross-channel and spatial interactions, these modules enhance the feature extraction capabilities of the network. Next, we embed the CSAF mechanism in the neck of the network, which effectively addresses the shortcomings of the YOLO network in detecting small targets. The experiments are conducted on a public dataset, testing four typical obstacles. The results show that PC-CS-YOLO outperforms YOLO11 in terms of accuracy, recall, and mAP by 2%, 3.9%, and 1.5%, respectively. When evaluated on a GPU, the inference speed is only 20.6 ms, improving computation speed by 15.3 ms compared to YOLO11, thus meeting the real-time requirements for blind navigation systems [45]. PC-CS-YOLO overcomes the limitations of most existing models, enabling simultaneous detection of multiple obstacles, enhancing feature extraction and fusion capabilities, reducing computational redundancy, and improving detection speed, thereby effectively reducing the risk for visually impaired individuals during their travel.

## Figures and Tables

**Table 1:** Recognition rules for typical types of obstacles.

Typical Behaviors	Description	Example
Vehicles	Vehicles driving on the road	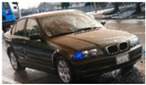
Pedestrian	Pedestrians walking on the side of the road	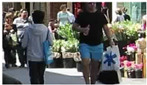
Traffic Cones	Signs on the pavement barrel obstruction	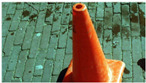
Road Surface Defects	Damaged, uneven, and cracked pavement	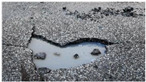

**Table 2:** Dataset of obstacles.

Types	Instances of the Training Set	Instances of the Validation Set	Instances of the Test Set	Total
Pedestrians	17,686	2286	2346	22,318
Vehicles	43,550	4953	5253	53,756
Traffic Cones	977	117	82	1176
Surface Defects	1482	115	122	1719

**Table 3:** The training parameters for the experiments.

Hyperparameters	Value
Optimization	SGD
Learning rate	0.01
Momentum	0.937
Weight decay	0.0005
Batch size	64
Warm-up epochs	3
Warm-up momentum	0.8
Warm-up deviation	0.1
Intersection over Union	0.7
Training epochs	200

**Table 4:** Result of ablation experiments based on PC-CS-YOLO.

Model	PCFN	CSAF	Precision (%)	Recall (%)	mAP50 (%)
YOLO11			81.7	75.8	82.6
YOLO11 + PCFN	✓		83.4	77.2	82.9
YOLO11 + CSAF		✓	83.1	79.6	83.1
PC-CS-YOLO	✓	✓	83.7	79.7	84.1

Note: ‘✓’ indicates that a corresponding improvement has been made.

**Table 5:** Run-time performance on the obstacle dataset.

Model	Processing Time
YOLO11	35.9 ms
PC-CS-YOLO	20.6 ms

**Table 6:** The results of obstacle detection for different models.

Model	Precision (%)	Recall (%)	mAP50 (%)
EfficientDet	84.5	74.2	82.0
Faster R-CNN	76.2	80.3	81.1
YOLOv5	80.4	77.1	81.9
YOLO11	81.7	75.8	82.6
D-FINE	82.3	76.9	82.8
PC-CS-YOLO	83.7	79.7	84.1

## Data Availability

Data will be made available on request.
